# Angiomatoid Fibrous Histiocytoma Initially Misdiagnosed as Elastofibroma Dorsi: A Case Report and Literature Review

**DOI:** 10.3390/medicina60111762

**Published:** 2024-10-27

**Authors:** Soyeon Jung

**Affiliations:** Department of Plastic and Reconstructive Surgery, Dongtan Sacred Heart Hospital, Hallym University College of Medicine, 7 Keunjaebong-gil, Hwaseong-si 18450, Gyeonggi-do, Republic of Korea; ps.soyeon.jung@hallym.or.kr; Tel.: +82-31-80862300; Fax: +82-31-80862258

**Keywords:** soft-tissue tumor, angiomatoid fibrous histiocytoma, elastofibroma dorsi

## Abstract

*Background and Objectives*: Angiomatoid fibrous histiocytoma (AFH) is a rare soft-tissue tumor with a low-grade malignancy. It typically arises in superficial soft tissues of the extremities, head, neck and trunk in children or young adults. Because of its rare entity, it tends to be confused and misdiagnosed. *Materials and Methods*: A 12-year-old male presented with a painless mass located on his right upper back. The CT finding showed a 7.3 × 2.8 × 5.4 cm-sized, well-defined heterogeneous soft tissue mass in the right infrascapular area. We performed a complete excision, including the surrounding capsule. *Result*: The final pathology revealed an AFH of intermediate malignancy. On pathologic examination, the lesion was a 5.8 × 4.5 × 2.6 cm-sized mass with a mitotic count of 12/10 HPF, tumor necrosis of 0% and marked increased cellularity and spindle cell morphology. The immunohistochemical study showed negative for S100 and positive for SMA, focal positive for Ki-67, CD68 and positive for CD99, Desmin staining. During the five years of follow-up period, he did not show any evidence of recurrence. *Conclusions*: The result was satisfactory. We report a case of AFH of the back initially misdiagnosed as an elastofibroma dorsi (ED) with the review of the literature for this uncommon entity.

## 1. Introduction

Angiomatoid fibrous histiocytoma (AFH) is a rare soft tissue tumor with low-grade malignant potential, commonly developing in the superficial soft tissues of the extremities and trunk in young ages [1]. Its incidence is approximately 0.3% of mesenchymal tumors [2]. Most cases are indolent and painless and appear in the extremities, trunk, head and neck. Besides the histological features specifically present an outer shell of lymphoid tissue, multinodular aggregates of dendritic-like tumor cells, blood-filled spaces and abundant admixed plasma cells, whereas unusual features were also observed in minor cases. They also include clear cells, rhabdomyoblast-like cells, pulmonary edema-like patterns and tumor cell cords lying in a myxoid stroma [3,4]. Elastofibroma dorsi (ED) is another soft benign tissue tumor with great rarity, accounting for 1–2% of the chest wall tumors. It also shows a similar presentation to AFH with non-tenderness and slow-growing [5,6]. It is typically found in the area of the back, which is the lower scapular region, more specifically. Whereas ED is usually found in elderly women and manual workers [7]. We report a case of AFH on the back, which was initially misdiagnosed as ED with a review of the literature. The case also illustrates the difficulty of the diagnostic approach to AFH. An appropriate permission and informed consent for publication were obtained from the patient.

## 2. Case Description

**Figure 1:**
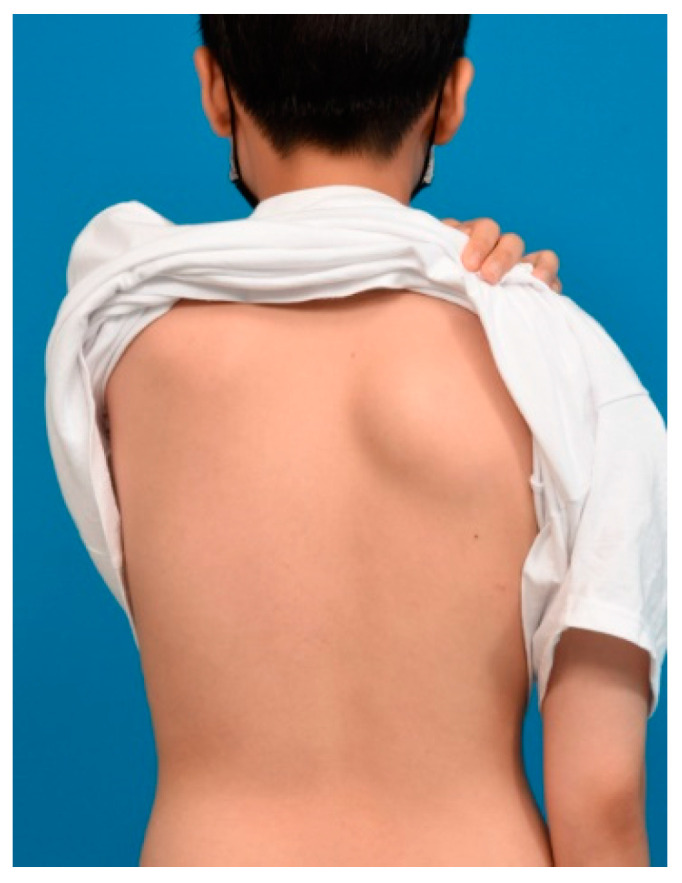
A 12-year-old male patient with prominent soft tissue mass on his right lower scapula.

**Figure 2:**
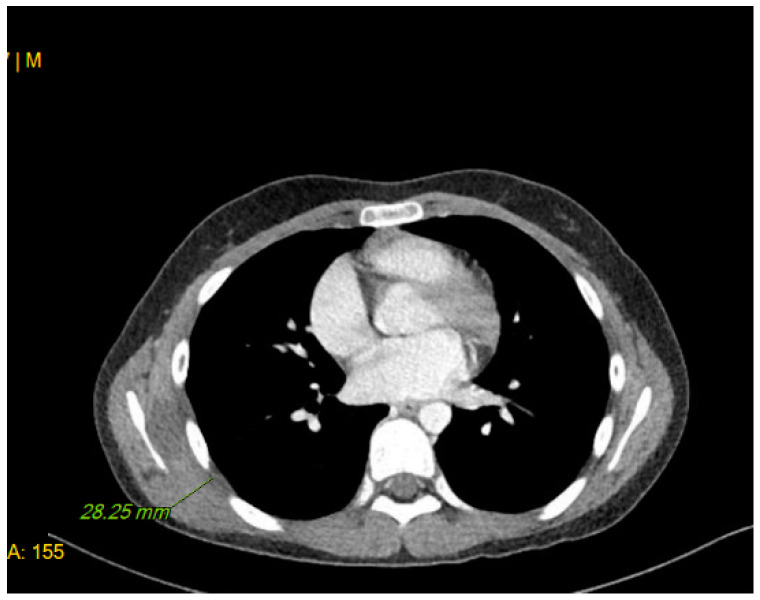
The image of CT scanning of the scapular mass initially presented list of differential diagnosis, elatofibroma dorsi (ED), vascular malformation or other benign soft tissue tumor.

**Figure 3:**
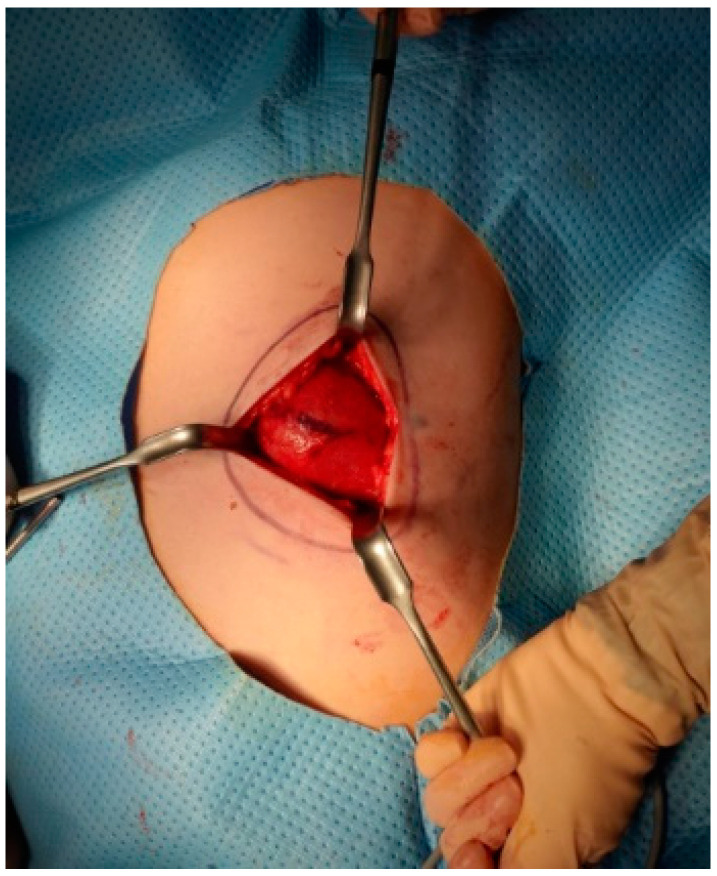
An intraoperative photograph of the surgical excision of the mass. The mass was seated deeply to the latissimus dorsi muscle.

**Figure 4:**
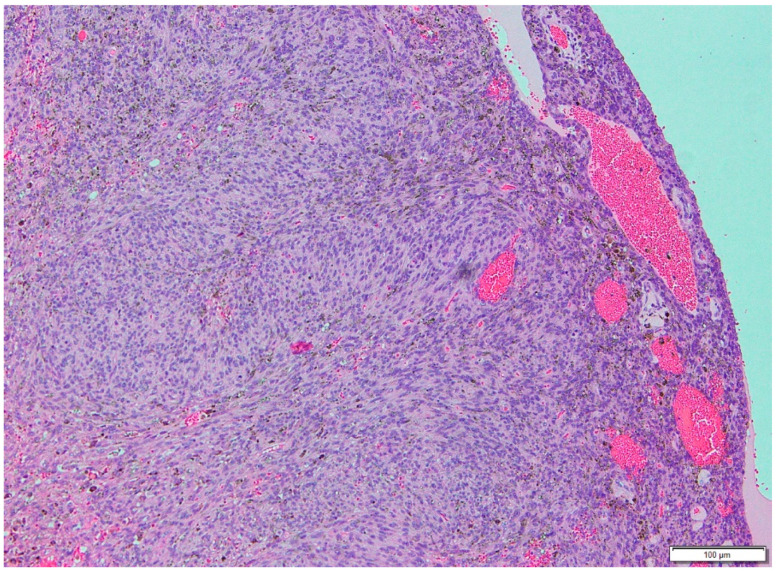
Hematoxlyn-Eosin staning showing spindle cells.

**Figure 5:**
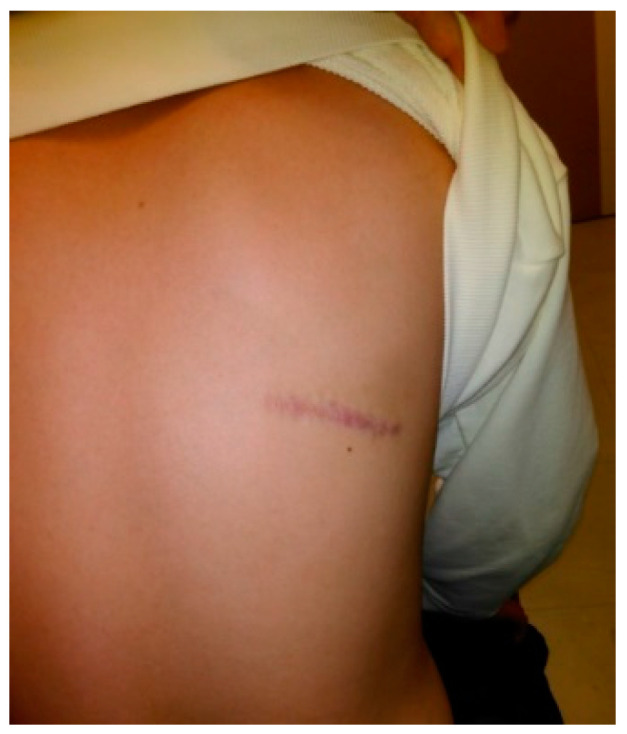
A postoperative photograph, two years observation.

A 12-year-old male presented with a painless, well-circumscribed mass located on his right upper back, which developed 9 months ago. Physical examination revealed a solid mass that became manifest on the abducting shoulder (Figure 1). The lesion was grossly rounded, of rubber-like consistency, not adhering to the skin but adhesive to deep structures. The computed tomography (CT) finding showed a 7.3 × 2.8 × 5.4 cm-sized, well-defined heterogeneous soft tissue mass in his right infra-scapular area. The mass was situated deeper than latissimus dorsi muscle and had no definite fatty component. There was neither significant lymph node enlargement in the mediastinum nor remarkable finding in the tracheobronchial tree and bony thorax observed. The initial report of differential diagnosis revealed elastofibroma dorsi (ED), vascular malformation or other benign soft tissue tumor (Figure 2). Under the impression of ED, we performed a complete excision, including the surrounding capsule. The surgical approach was started to incise the skin along with the skin crease of the infra-scapular area. The dissection was continued to the deep plane below the latissimus dorsi muscle, and the sizable mass was exposed (Figure 3). Microscopically, the lesion was encapsulated with yellowish tissue of fatty consistency. The defect following the mass excision was closed in layers with surrounding tissue repair, including muscle. The skin envelope was primarily closed with minimal tension. The procedure was smooth and uneventful. The post-operative recovery from surgery was not complicated, and the patient was discharged the next day. The final pathology confirmed an Angiomatoid fibrous histiocytoma (AFH) of intermediate malignancy. From the pathologic examination, the lesion was 5.8 × 4.5 × 2.6 cm-sized, subsequently, it found a mitotic count of 12/10 HPF, tumor necrosis of 0% and marked increased cellularity and spindle cell morphology. The following immunohistochemical study showed negative for S100 and positive for SMA, focal positive for Ki-67, CD68 and positive for CD99, Desmin staining (Figure 4). He visited the medical office every 6 months for serial clinical examinations. During the five years of follow-up period, he did not have any evidence of recurrence (Figure 5).

## 3. Discussion

Angiomatoid fibrous histiocytoma (AFH) is a rare soft tissue tumor of uncertain differentiation. It develops in the superficial layer of the extremities, trunk, head and neck in the first two decades of life [4,5]. AFH was initially reported as diverse types of malignant fibrous histiocytoma; however, the World Health Organization formally removed AFH as a subtype of malignant sarcoma in 2002. Instead, it was classified into the category of tumors with uncertain differentiation in 2013 [6]. According to the previous reports, it is a more likely benign tumor but still shows a low-grade of malignancy [5,6]. Despite this reclassification, the exact category of the tumor and its differentiation for AFH is not clear yet. Mostly patients rarely complain of painful mass; however, some suffer from symptomatic mass and nodules [7,8]. Pathologic consultation is essential in the differential diagnosis of AFH. The tumors are grossly well-circumscribed and enclosed by a fibrous pseudo-capsule. It occurs in a wide morphologic spectrum but consistently shows the critical features such as multi-lobular growth of round cells, spindle cells or histiocyte-like cells with low mitotic activity. Furthermore, inflammatory infiltrates containing lymphocytes and plasma cells surround it [8,9]. When considering these typical development and presentation, the case we experienced showed similar features in principle. However, minor differences like the plane of the mass that was situated, which was a bit deeper than the occasions’ in most cases. As far as we know, its non-specific presentation and radiologic findings make it more difficult to distinguish between one and another.

Genetically, AFH is frequently associated with the three fusion-genes, such as EWSR1-CREB1, EWSR1-ATF1, and the FUS-ATF1 fusion gene [10,11,12]. Among them, EWSR1-CREB1 is the most found in AFH [10]. Despite the genetic analysis, some tumors sharing those genetic fusions exist, and they still cause diagnostic difficulties [11,12]. Similarly to this case, Michcik et al. also reported a case highlighting the difficulty of discovering AFH [13]. Various diagnostic modalities were employed; nevertheless, the diagnostic confirmation was not achieved. A venous malformation was only aroused, and the surgical excision was followed by. Eventually, the genetic test was performed, and the final diagnosis of AFH was revealed [13].

Elastofibroma dorsi (ED) is an infrequent soft tissue benign tumor with indolent fashion. It was defined in 2002 by the World Health Organization (WHO) soft tissue’s tumors taxonomy as a benign fibroblastic/myofibroblastic tumor [14]. Unlike AFH, ED affects primarily the elderly over 55 years of age, with a mean age of 60 years. Nevertheless, some cases of ED have been described also in children. ED is found more frequently in women than men. Although ED is rare and indolent, a medical practitioner should be suspicious of the tumor when a patient complains of discomfort in response to scapula movement [15]. There are three major theories that explain etiology. The first is chronic and repetitive mechanical stresses that lead to microtrauma, which continues overproduction of elastic tissue from the stimulated fibroblasts. The second one is reactive fibromatosis and secondary degeneration of elastic fibers due to vascular insufficiency, which tends to affect elderly women. The last theory is a familial predisposition with an underlying enzymatic disorder or defect [14,15]. When considering that ED is rarely mentioned in the wide spectrum of differential diagnosis of AFH, this case illustrates the challenges associated with reaching the accurate diagnosis [10].

Both Angiomatoid fibrous histiocytoma (AFH) and Elastofibroma dorsi (ED) are rare and indistinct clinical entities. Basically, it is hard to reach the certainty for the initial diagnosis with physical examination, past history, complained symptoms, or even imaging studies. However, the most pivotal imaging tool should be magnetic resonance (MR) scanning among the diverse modalities from our experience and reviewing the previous literature [14]. Nevertheless, molecular studies play a vital role in verifying angiomatoid fibrous histiocytoma (AFH); thus, immunohistochemistry demonstrates positivity for desmin, CD68, and CD99, whereas cytogenetic evaluation demonstrates that the EWSR1-CREB1 fusion gene is present in a majority of AFH cases as addressed above [16,17]. Because of the lack of a specific immunoprofile, some misdiagnosis or misclassification of the tumor may occur, and it can lead to wrong management or treatment [12]. Although most cases of AFH have shown a favorable prognosis; early suspicion is essential to establishing the correct treatment plan considering the risk of local recurrence and metastasis. Thus, it is believed that appropriate wide excision and tumor-free margin under widespread impression should be critical. In addition, follow-up treatment and close observation are equally important.

Misdiagnosis originates from the rarity of prevalence and the similarity of clinical presentations. Basically, misdiagnosed lesions are frequently confused with mimicking lesions because of their non-specific symptoms and signs. Thus, some are challenged to unveil the tumor identification even though the careful inspection and examination with imaging modalities were conducted [18,19]. The occasions we should pay more attention to approach the suspecting tumors exist. For instance, the tumor developed in childhood can show atypical presentations, and it leads to a delayed diagnosis and surgical treatment. The delayed intervention cannot promise the best result. Therefore, when encountering the lesions with uncertainty in children, an early biopsy is essential to have an opportunity for a better prognosis [20]. In addition, there are the malignant lesions with some chances to be confused with another tumor. Cassalia et al. also reported the comprehensive review of misdiagnosis in skin and soft tissue cancer [21,22]. When considering the importance of accurate and early diagnosis in the treatment of malignant tumors, the high level of suspicion and an early biopsy are critical to excluding malignancy. Eventually, those mimicking tumors should require the immunohistochemistry or genetic analysis to be differentiated after surgical excision. Similarly, previous published papers about the mimicking tumors and challenges of diagnosis elucidated the need for further evaluations such as immunohistochemistry or genetic studies beyond the simple microscopic examination.

## 4. Conclusions

Angiomatoid fibrous histiocytoma (AFH) is an uncommon, low-grade malignant tumor. It presents non-specific findings in clinical examination and imaging studies. Especially, its presentation is similar to the clinical features of elastofibroma dorsi (ED). Thus, an imaging study is required to speculate the mass. Among the imaging modalities, MR scanning might be more valuable than others for differential diagnosis. Complete surgical excision is the therapeutic option for this tumor eventually. Above all, pathological study, immunohistochemical test, and further genetic analysis should be required to differentiate and verify the tumor after surgery.

## Data Availability

The data presented in this study area available on request from the corresponding author due to privacy.
